# Metastasis in the gallbladder: does literature reflect reality?

**DOI:** 10.1007/s00428-022-03314-7

**Published:** 2022-03-31

**Authors:** Tessa J. J. de Bitter, Daan M. Trapman, Femke Simmer, Niek Hugen, Elise A. J. de Savornin Lohman, Philip R. de Reuver, Joanne Verheij, Iris D. Nagtegaal, Rachel S. van der Post

**Affiliations:** 1 Radboud Institute for Molecular Life Sciences, Department of Pathology, Radboud University Medical Center, P.O. Box 9101, Nijmegen, 6500 HB The Netherlands; 2 Radboud Institute for Health Sciences, Department of Surgery, Radboud University Medical Center, Nijmegen, 6500 HB The Netherlands; 3 Department of Surgery, Rijnstate Hospital, Arnhem, 6815 AD The Netherlands; 4 Cancer Center Amsterdam, Amsterdam UMC, Department of Pathology, University of Amsterdam, Amsterdam, The Netherlands

**Keywords:** Gallbladder cancer, Metastasis, Colorectal cancer, Melanoma, Renal cell carcinoma

## Abstract

**Background:**

Metastases to the gallbladder (GBm) are rare and pose a unique diagnostic challenge because they can mimic a second primary tumor. This study aimed to gain insight into the clinicopathological and epidemiological characteristics of GBm.

**Methods:**

A comprehensive literature review was performed (literature cohort) and compared with a nationwide cohort of GBm patients diagnosed between 1999 and 2015 in the Netherlands, collected via two linked registries (population cohort). Overall survival (OS) was estimated by Kaplan–Meier. Hazard ratios were determined by a Cox proportional hazard model.

**Results:**

The literature cohort and population cohort consisted of 225 and 291 patients, respectively. In the literature cohort, melanoma was the most frequent origin (33.8%), while colorectal cancer was the most frequent origin in the population cohort (23.7%). Prognosis was poor with median OS ranging from 6.0 to 22.5 months in the literature and population cohorts, respectively. Age, timing of GBm (synchronous/metachronous) and primary tumor origin were independent prognostic factors for OS.

**Discussion:**

Metastases to the gallbladder are rare and carry a poor prognosis. Differences between both cohorts can be attributable to the biased reporting of tumor types that are more easily recognized as GBm because of distinct histological features.

**Supplementary Information:**

The online version contains supplementary material available at 10.1007/s00428-022-03314-7.

## Introduction

Metastatic spread accounts for 90% of all cancer-related deaths [1], but remains poorly understood. Rare metastatic sites pose new diagnostic challenges because less recognized and their impact is unknown.

Large-scale autopsy studies have provided preliminary insight into metastatic patterns and showed that preferential metastatic sites vary greatly across different primary tumors [2,3]. For example, colorectal cancer most frequently metastasizes to the liver and lung, while breast and lung cancers can metastasize to multiple organs including bone, brain and liver. Some organs are rarely affected by metastatic spread, including the gallbladder. Metastasis to the gallbladder (GBm) was found in merely 2.2% to 5.8% in patients with metastatic cancer in two autopsy studies [2,3]. In both studies, gastric cancer was the most common primary origin of GBm in 5.7% and 14.3% of cases, whereas other common primaries were breast cancer (4.4%) ^[[[[[2]]]]]^ and pancreatic cancer (12.5%) [3].

A more recent Korean study [4] focused on computed tomography (CT) features of GBm from various primary origins. Also in this study, with 21 cases, gastric cancer was the most frequent primary origin (38.1%). Gastric cancer, however, shows marked geographic variation, with a high incidence in Korea, and therefore, frequencies might not easily be extrapolated to other geographic regions.

The sensitivity of current imaging modalities for primary GBC is poor, and GBm cannot be distinguished from primary gallbladder cancer based on CT features [4]. In addition, we recently showed that after surgery for presumed primary GBC, GBm may go unnoticed because it can mimic a second primary tumor. Two out of ten patients that were initially diagnosed with primary GBC by the pathologist [5] appeared to have GBm from colorectal origin based on molecular clonality analysis.

Evidently, better insight into the clinicopathological characteristics of GBm is needed to improve timely detection, treatment and prognosis. To this end, a comprehensive literature search was performed to identify all cases with GBm presented in the literature. As no reliable nationwide or population-based data were available in the literature, findings were compared with data of a nationwide cohort of patients diagnosed with GBm in the Netherlands.

## Methods

### Literature cohort

**Fig. 1:**
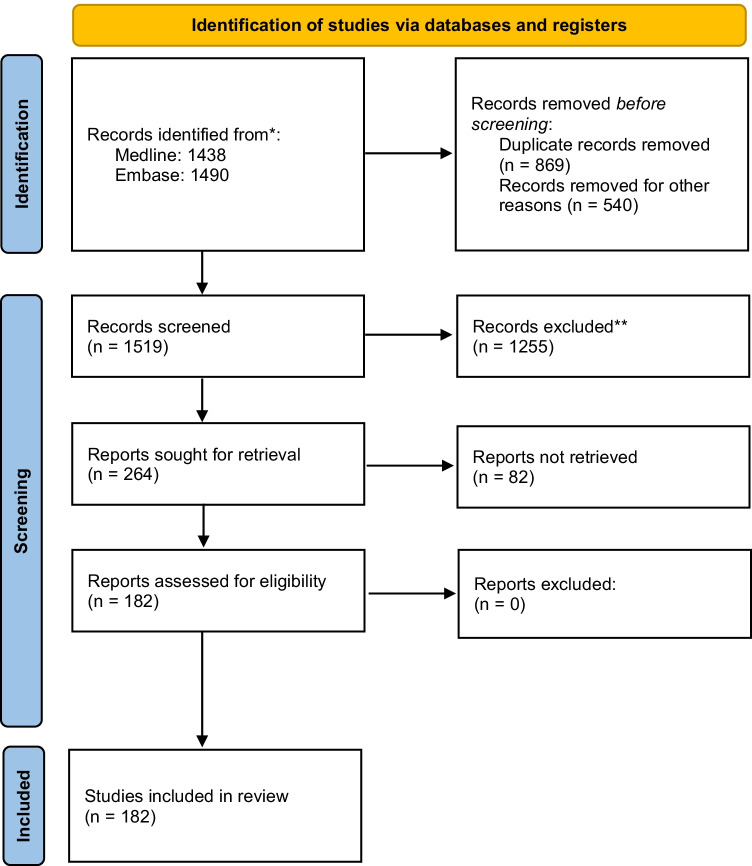
Flowchart of the literature search

The literature cohort was based on a systematic search of Medline and Embase on the Ovid platform on February 2, 2021, to identify all cases with GBm. No restrictions were made to publication date. The search strategy is shown in Fig. 1.

References were imported in EndNote (version X9.0.1, Clarivate Analytics, Philadelphia, PA, USA), and duplicates were removed. For the remaining references, two independent researchers reviewed title and abstract and excluded all articles not fulfilling the following inclusion criteria: case report or retrospective study describing clinical case(s) of GBm from any cancer type (Fig. 1). For articles without an abstract, the full text was accessed to evaluate if they met inclusion criteria.

### Population cohort

Data from a nationwide retrospective cohort of patients with a gallbladder tumor between 1991 and 2015 and a history of other malignancies were collected as described before [6] using the Nationwide Network and Registry of Histopathology and Cytopathology in the Netherlands (PALGA, LZV-1152) [7] and the Netherlands Cancer Registry (NCR, K14.142). Cases were manually checked and verified for inclusion (i.e., when GBm diagnosis was mentioned in conclusion section of the pathology report).

### Data extraction

Data on gender, age at diagnosis of GBm, time interval between diagnosis of the primary tumor and development of GBm, vital status and follow-up, type and histology of the primary tumor and additional pathological findings were extracted from the literature and the population cohort.

### Statistical analyses

Patient and tumor characteristics were described using counts and percentages. The crude incidence rate of GBm between 1991 and 2015 (population cohort) was estimated by dividing the annual incidence of GBm by the annual incidence of all solid cancer types (population at risk) in the Netherlands. The annual cancer incidence was derived from the NCR [8].

For survival analyses, overall survival (OS) was defined as the interval in months between GBm diagnosis and time of death or last follow-up (February 1, 2020). Patients alive at the last date of follow-up were censored. Survival curves were made according to the Kaplan–Meier method. All primary cancer sites for which the incidence was < 10 were categorized as “other.”

Multivariate survival analysis was performed using the Cox proportional hazard model, and log minus plots were used to assess whether the proportional hazards assumption was met. No significant violations were observed that required the use of a time-dependent Cox proportional hazard model.

All tests of significance were two-tailed, and P-values of < 0.05 were considered statistically significant. Statistical analyses were performed with IBM SPSS Statistics (version 22.0.0.1, IBM, Armonk, NY, USA).

## Results

### Patient characteristics

#### Literature cohort

In the literature cohort, 178 case reports and 4 retrospective case series were included (Fig. 1 and Supplemental Table 1), adding up to 225 patients. Over half of the patients were male (56.0%), and the median age at GBm diagnosis was 61 years (SD ± 14.6 years). About half of the patients (45.8%) presented with isolated metastasis to the gallbladder. The other patients presented with multiple metastases at time of GBm diagnosis (44.4%) or during follow-up (9.8%).

#### Population cohort

The population cohort entailed 291 patients diagnosed with GBm between 1991 and 2015 in the Netherlands. About half of the patients were male (47.5%), and the median age at GBm diagnosis was 65 years (SD ± 13 years). The majority of patients presented with isolated metastasis to the gallbladder without a histologically proven history of metastatic disease elsewhere (70.1%).

### Incidence of GBm over time

#### Population cohort

**Fig. 2:**
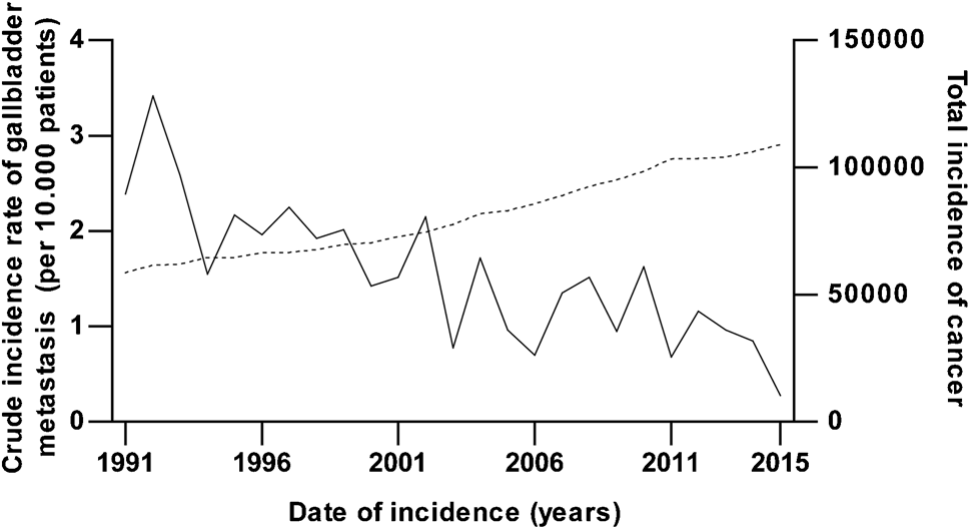
Crude incidence rate of gallbladder metastasis per 10,000 cancer patients (solid line) compared with the total number of cancer patients (dashed line), population cohort

Annually, between 3 and 21 patients were diagnosed with GBm in the population cohort. The crude incidence rate of GBm decreased from 1.3/10000 cancer cases in 1991 to 0.3/10000 in 2015 in the Netherlands (Fig. 2).

### Origin of GBm

#### Literature cohort

**Fig. 3:**
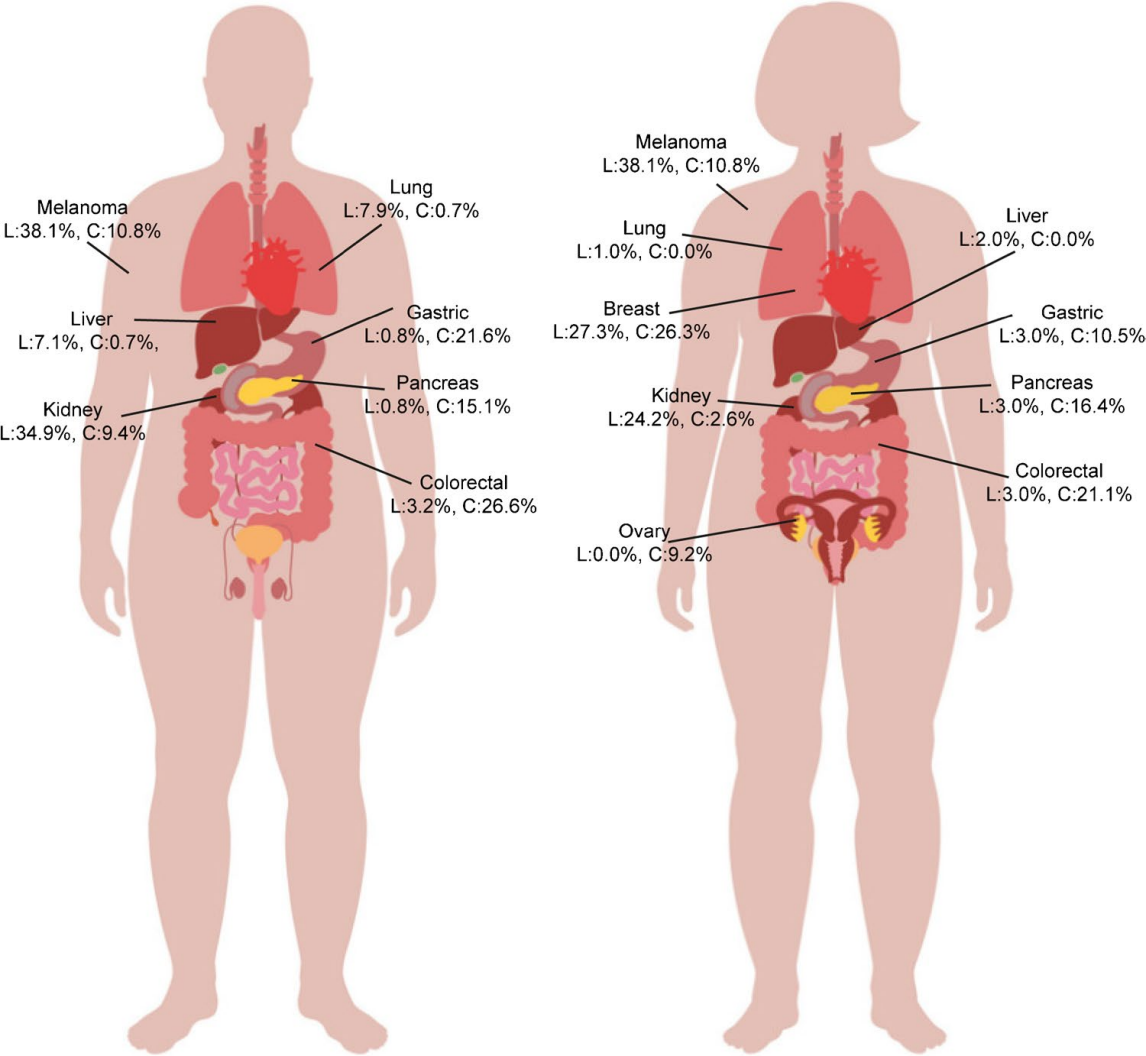
Distribution of gallbladder metastasis cases according to primary tumor origin. **A**, male patients; **B**, female patients. Only primary origins for which ≥ 10 cases were reported in either the clinical or literature cohort were included. **L**: literature cohort; **C**: population cohort

In the literature cohort, the most frequently reported primary origin was melanoma (males: 38.1%, females: 28.3%), followed by renal cell carcinoma (males: 34.9%, females: 24.2%). In females, breast cancer was the most frequent primary origin (27.3%) (Fig. 3, Supplemental Table 2).

#### Population cohort

In the population cohort, primary tumors most frequently originated from the gastrointestinal tract (61.5%), including colorectal cancer (males: 26.6%, females: 21.1%), gastric cancer (males: 21.6%, females: 10.5%) and pancreatic cancer (males: 15.1%, females: 16.4%) (Fig. 3, Supplemental Table 3). In line with the literature cohort, in the population cohort breast cancer was the most frequent primary origin of GBm in females (26.3%).

The histological type of primary breast cancer was mainly invasive ductal carcinoma/adenocarcinoma not otherwise specified (NOS) (64.3%). In addition, invasive lobular breast cancer was frequently observed (35.7%). If the primary tumor originated from colorectal cancer, the histological type of the majority of cases was adenocarcinoma NOS (83.8%). Mucinous adenocarcinoma was found in 14.7% of cases and neuroendocrine carcinoma in 1.5% of cases with CRC as the primary origin. The histology of gastric cancer patients consisted primarily of adenocarcinoma NOS (65.2%), followed by signet ring cell carcinoma (32.6%) and mucinous adenocarcinoma (2.2%). Nearly all pancreatic cancer cases were adenocarcinoma (97.8%), and in only one case, a neuroendocrine carcinoma was found (2.2%). For the literature cohort, data on histological subtype were missing in the majority of cases.

### Timing of GBm

#### Literature cohort

**Fig. 4:**
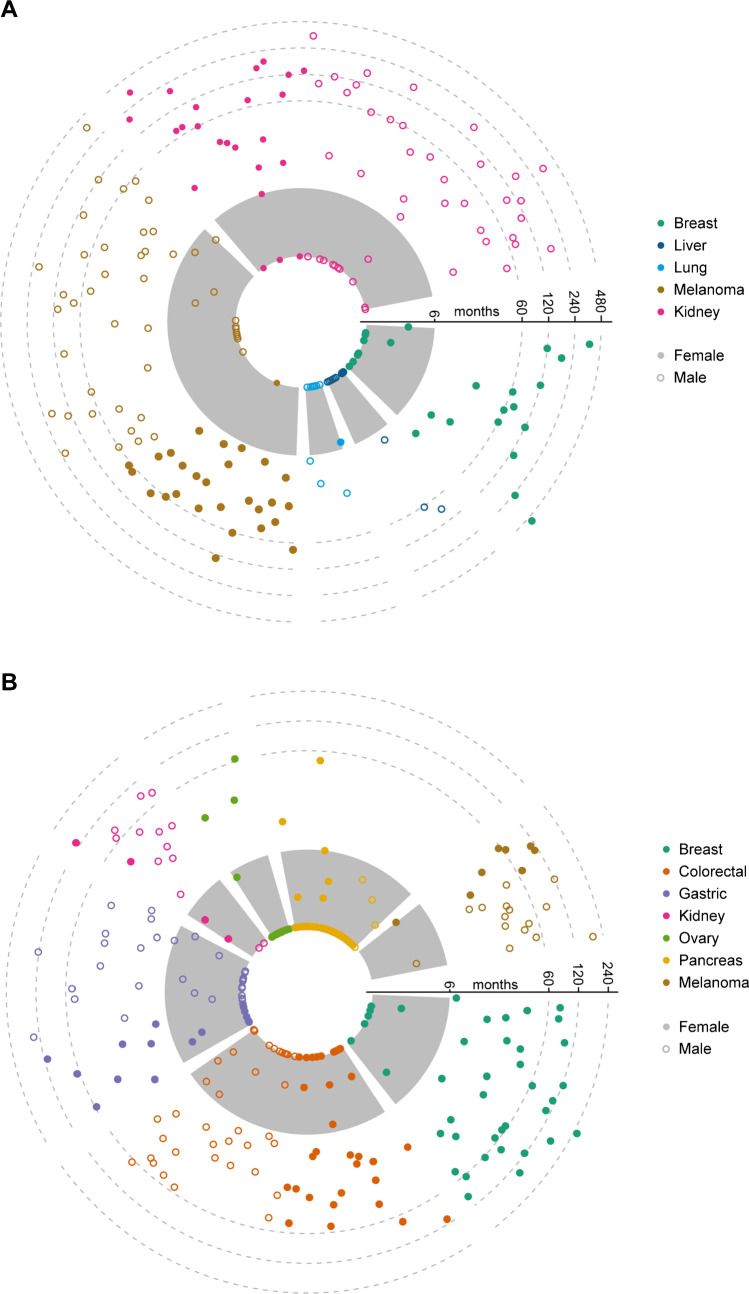
Timing of GBm. Interval in months between primary tumor and gallbladder metastasis diagnosis, according to primary origin (log10 scale). **A**, literature cohort; **B**, population cohort. Primary tumor origins were only included if N ≥ 10. Gray shading marks the synchronous metastases interval (6 months)

In the literature cohort, the majority of GBm cases was diagnosed more than six months after primary tumor diagnosis (metachronous) (66.2%). The interval between primary tumor diagnosis and GBm varied according to primary site (Fig. 4, Supplemental Table 2). Largest intervals between primary cancer and GBm diagnosis were observed for renal cell carcinoma, melanoma and breast cancer (females only) with mean intervals of 72.3, 47.6 and 86.2 months, respectively.

#### Population cohort

In the population cohort, metachronous GBm was diagnosed in 51.9% of cases (Fig. 4, Supplemental Table 3). Intervals were shorter than in the literature cohort, with the largest GBm. Intervals were observed for melanoma, renal cell carcinoma and breast cancer (females only) with mean intervals of 53.3, 39.3 and 54.5 months, respectively. For pancreatic cancer, however, GBm was mainly diagnosed synchronously (i.e., within six months after primary tumor diagnosis) with a mean interval of 2.0 months.

### Survival

#### Literature cohort

**Fig. 5:**
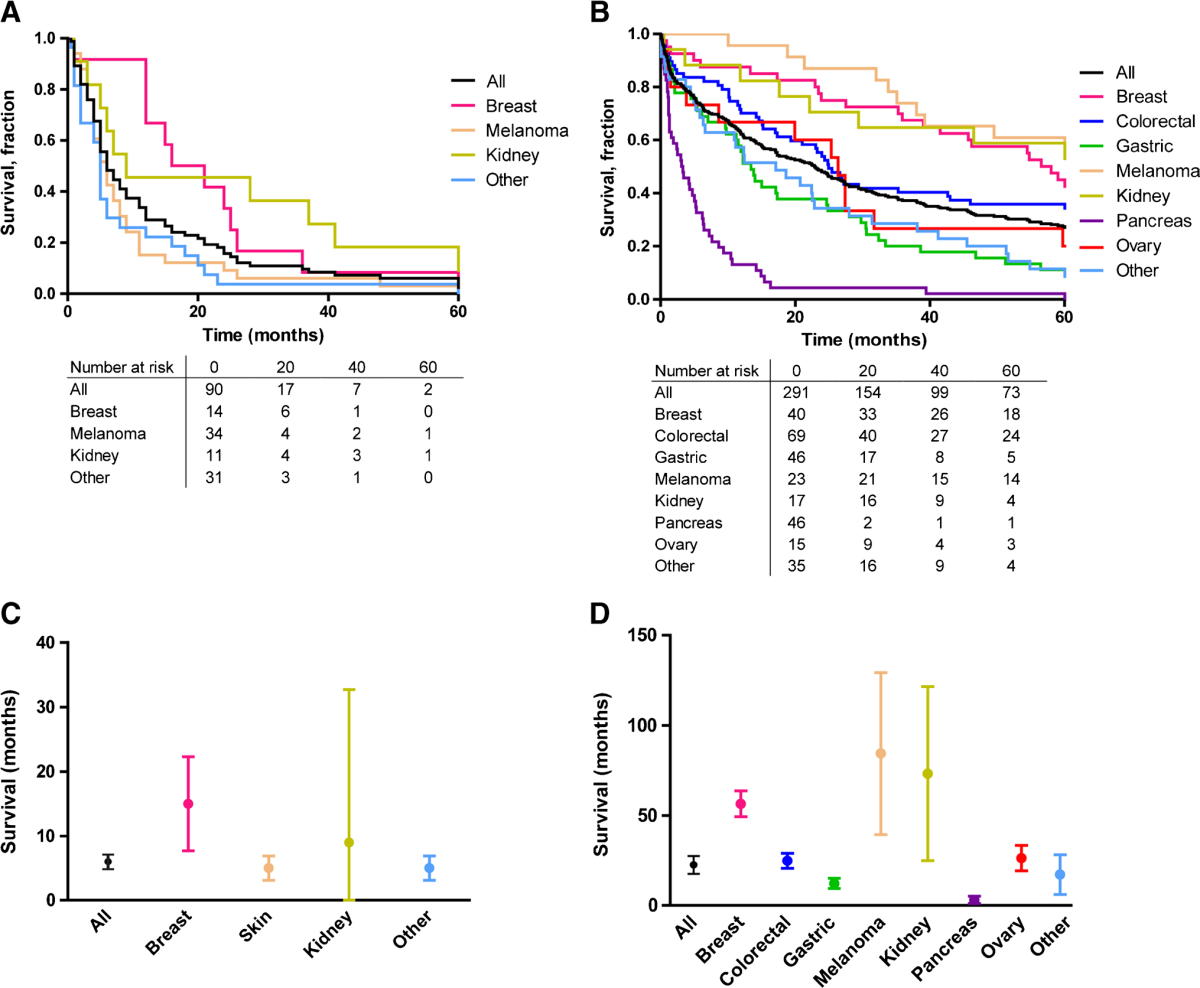
Overall survival of metastatic gallbladder cancer according to primary tumor location. Survival was measured from date of diagnosis of gallbladder metastasis. Primary origins for which N < 10 were grouped under “other.” **A**, literature cohort; **B**, population cohort. **C**-**D**, median overall survival rates with 95% confidence intervals according to primary tumor location. **C**, literature cohort; **D**, population cohort

The median OS for patients with GBm was poor with 6.0 months (95% CI 4.8–7.1) in the literature cohort (Fig. 5). The worst OS was observed in patients with a primary melanoma (median OS 5.0 months, 95% CI 3.1–6.9). Patients with GBm originating from breast cancer had the longest OS (median 15 months, 95% CI 7.7–22.3).

#### Population cohort

The median OS for patients with GBm in the population cohort was 22.5 months (95% CI 17.5–27.5) (Fig. 5). The shortest OS was observed in patients with a primary tumor originating from the pancreas (median OS 3.1 months, 95% CI 1.1–5.2). In contrast to the literature cohort, patients with GBm originating from melanoma had the longest survival population cohort(median OS 84.4 months, 95% CI: 39.5–129.2).

Age at GBm diagnosis, timing of GBm diagnosis (synchronous/metachronous) and primary tumor origin were independent prognostic factors for OS (Table 1).

**Table 1:** Multivariate survival analysis of the population cohort (Cox proportional hazard model)

Variable	No. of patients	Hazard ratio	95% confidence interval
Age at diagnosis			
< 60	114	1	
60–74	74	1.15	0.82—1.61
≥ 75	103	2.16	**1.6—2.92**
Gender			
Male	139	1	
Female	152	0.90	0.68—1.19
Timing of gallbladder metastasis			
Synchronous	151	1	
Metachronous	140	0.31	**0.22—0.42**
Isolated or multiple metastases			
Isolated	204	1	
Multiple	87	1.3	0.96—1.77
Primary tumor origin			
Colorectal	69	1	
Gastric	46	1.81	**1.2—2.72**
Pancreas	46	3.19	**2.07—4.92**
Breast	40	0.97	0.61—1.54
Melanoma	23	0.78	0.43—1.4
Kidney	17	0.68	0.36—1.25
Ovary	15	0.88	0.46—1.67
Other	35	1.04	0.65—1.67

In bold: significant prognostic factors for OS

## Discussion

Metastases to the gallbladder are rare and poorly understood. This is the first study providing insight into the clinicopathological characteristics of GBm from a comprehensive literature review and a population-based perspective. It shows that GBm can develop from various types of primary tumors, that the interval between primary and mGCB diagnosis is highly variable and that the outcome of GBm is generally poor.

Over a time period of 14 years (1991–2015), the crude incidence rate of GBm decreased, while an increase in the total number of cancer patients was observed. The crude incidence rate of GBm in this study is likely an underestimation, since only patients with pathology-confirmed diagnosis were included. Tissue sampling of the gallbladder is challenging because of its anatomical location, and a cholecystectomy is a relatively invasive procedure to confirm metastatic disease. With the wide availability of advanced imaging techniques such as PET/CT, other metastatic sites outside the gallbladder might be more easily detected and biopsied to diagnose metastatic disease.

The most notable difference between the literature cohort and population cohort was the primary origin. In the literature cohort, the most frequently observed primaries were melanoma and renal cell carcinoma. In the population cohort, however, GBm mostly originated from tumors of the gastrointestinal tract, including colorectal cancer, gastric cancer and pancreatic cancer. Two relatively large retrospective case series of melanoma and renal cell carcinoma that were included in the literature cohort could have skewed the data [9,10]. In addition, both cancer types show distinct histological features that directly point toward an origin outside the gallbladder. We and others showed before that specifically metastatic (gastrointestinal) adenocarcinomas that display mucosal colonization can be misdiagnosed as second primary [5,11], which possibly resulted in reporting bias.

Distinction between primary gallbladder cancer and GBm is of particular importance for optimal therapy selection. Whereas primary gallbladder cancer may benefit from surgical resection, GBm generally requires a systemic approach, depending on its primary origin and extent of metastatic spread. However, patients with isolated metastases toward the gallbladder might benefit from surgical removal of the metastatic lesion. Morphology and immunohistochemical characteristics can be compared between the primary cancer and the gallbladder cancer, which may lead to a diagnosis in most cases. As routine histopathological assessment cannot reliably distinguish primary versus metastatic gallbladder cancer from colorectal metastases, molecular clonality analysis is advised for patients with gallbladder cancer and a history of gastrointestinal malignancies [5].

The interval between primary tumor and GBm diagnosis was heterogenous for both cohorts, but in general the gastrointestinal primaries more frequently resulted in synchronous metastases, whereas the non-gastrointestinal primaries more frequently resulted in metachronous metastases. This is most likely the result of the difference in proximity of primary and metastatic location, rather than preferential metastatic sites of different primaries [12].

Survival after GBm diagnosis was poor with 6.0 to 22.5 months in the literature cohort and population cohort, respectively. Primary tumor origins with the largest intervals between primary and GBm diagnosis (breast, melanoma, kidney) also had the best OS rates in the population cohort; gastric and pancreatic origins had the lowest OS rates.

A major strength of this study is the combined analysis of a large population cohort, representing real-life data, and an extensive literature review. Our data significantly contribute to the existing literature, which is limited to case reports and small case series, and provide more insight into the clinicopathological characteristics of GBm.

Some limitations should be addressed as well. First, only pathology-confirmed GBm diagnoses were included. This likely resulted in an underestimation of the incidence of GBm. In addition, there might be a selection bias with inclusion of patients with limited or unsuspected metastatic disease. In addition, all clinicopathological data were retrospectively collected and cholecystectomy specimens were not reviewed. This may have resulted in diagnostic heterogeneity and misdiagnosis, specifically because diagnosis in some cases proved to be challenging ^[[[[[5]]]]]^. Third, although a comprehensive literature search was performed, the large differences in primary origins compared with those from the population cohort may point toward reporting bias of primary origins with a more distinct histological appearance.

In conclusion, metastasis toward the gallbladder is rare and gastrointestinal primary cancers predominate. The interval between primary tumor diagnosis and GBm diagnosis is origin dependent. Survival of patients with GBm is generally poor, but some primary origins (e.g., breast and kidney) showed relatively longer survival compared with others.

## Supplementary Information

Below is the link to the electronic supplementary material.

Supplementary file1 (XLSX 37 KB)

## Data Availability

Not applicable.

## References

[1] Chaffer CL, Weinberg RA (2011). A perspective on cancer cell metastasis. Science.

[2] Disibio G, French SW (2008). Metastatic patterns of cancers: results from a large autopsy study. Arch Pathol Lab Med.

[3] Abrams HL, Spiro R, Goldstein N (1950). Metastases in carcinoma; analysis of 1000 autopsied cases. Cancer.

[4] Choi WS (2014). CT findings of gallbladder metastases: emphasis on differences according to primary tumors. Korean J Radiol.

[5] de Bitter TJJ (2019). Colorectal metastasis to the gallbladder mimicking a primary gallbladder malignancy: histopathological and molecular characteristics. Histopathology.

[6] Hugen, N., et al (2021) Umbilical metastases: Real-world data shows abysmal outcome*.* Int J Cancer 10.1002/ijc.33684 PMC8361932 33990961

[7] Casparie M (2007). Pathology databanking and biobanking in The Netherlands, a central role for PALGA, the nationwide histopathology and cytopathology data network and archive. Cell Oncol.

[8] Netherlands Cancer Registry (2021) Accessed April 12, 2021.

[9] Dong XD (1999). Melanoma of the gallbladder: a review of cases seen at Duke University Medical Center. Cancer.

[10] Chung PH (2012). Renal cell carcinoma with metastases to the gallbladder: four cases from the National Cancer Institute (NCI) and review of the literature. Urol Oncol.

[11] Estrella JS (2011). Mucosal colonization by metastatic carcinoma in the gastrointestinal tract: a potential mimic of primary neoplasia. Am J Surg Pathol.

[12] Langley RR, Fidler IJ (2011). The seed and soil hypothesis revisited–the role of tumor-stroma interactions in metastasis to different organs. Int J Cancer.

